# Toxic environment and obesity pandemia: Is there a relationship?

**DOI:** 10.1186/1824-7288-36-8

**Published:** 2010-01-22

**Authors:** Giuseppe Latini, Francesco Gallo, Lorenzo Iughetti

**Affiliations:** 1 Division of Neonatology, Perrino Hospital, Brindisi, Italy; 2 Clinical Physiology Institute, National Research Council of Italy (IFC-CNR), Lecce Section, Italy; 3 Division of Pediatrics, Perrino Hospital, Brindisi, Italy; 4 Department of Paediatrics, University of Modena and Reggio Emilia, Modena, Italy

## Abstract

Obesity is a multi-factorial disease, resulting from genes, environment and behaviour interactions, and represents the most common metabolic disorder in the Western Hemisphere. Its prevalence has dramatically risen during the last three decades, reaching worldwide epidemic proportions. Recent cumulating evidence suggests that obesity may represent an adverse health consequence of exposure during the critical developmental windows to environmental chemicals disrupting endocrine function. Moreover, exposure to these chemicals seems to play a key role in the development of obesity-related metabolic and cardiovascular diseases. Further research is needed to elucidate the relationship between this exposure and the obesity pandemia and the involved mechanisms as well as to refine hazard identification.

## Introduction

Obesity has recently become of great interest, due to a significant increase of this pathology during the last three decades both in children and adults. In fact, obesity represents the most common metabolic disorder in the Western industrialized countries, even if it is reaching worldwide epidemic proportions involving also developing countries [1-4]. In addition, obesity is the major risk factor for the development of insulin resistance, believed to be an important link between obesity and the associated metabolic syndrome diseases, known to reduce quality and length of life and increase medical costs [5].

Since obesity is associated with sedentary lifestyle patterns and inadequate dieting behaviours, obesity has been thought to be explained by a prolonged positive energy balance. However, this idea is now being challenged, as several social, economic and environmental factors have been shown to influence human physical growth and development, and obesity is one of the many diseases shown to have a developmental origin. In particular, obesity may be increasing as a function of developmental nutrition and exposure to environmental chemicals during the critical early life period. These compounds interfere with the body's adipose tissue biology and interact with hormone receptors. They mimic or antagonize the actions of endogenous hormones, thus disrupting the programming of endocrine signalling pathways during critical windows of early development and differentiation. As a consequence, they are commonly defined as endocrine disrupting chemicals (EDC). Infants and children may be considered a highly susceptible population to EDC exposures [6-13]. At this regard, it should be underlined that the adipose tissue is an important endocrine tissue secreting obesity/diabetes-related hormones and cytokines [14]. Developmental exposure to EDC can create abnormalities within the homeostatic control systems required to maintain a normal body weight throughout life [15]. In addition, recent evidence suggests that EDC may alter mechanisms involved in weight homeostasis, with consequent weight gain by increased volume of adipose tissue [16]. Moreover, lipophilic environmental pollutants, including persistent organic pollutants (POPs), pesticides, polychlorinated biphenyls (PCBs) and polybrominated diphenyl ethers (PBDEs) have been shown to accumulate in adipose tissue after exposure. On the other hand, increased adipose tissue results in altered storage of lipophilic toxicants, that may disregulate the cytochromes p450 gene expression profile in rat white adipose tissue [14,17].

Finally, it has been hypothesized that not only environmental toxicants can modulate the genes expression, but can also produce epigenetic effects, affecting in an inheritable manner the metabolic status in humans [18,19].

## Discussion

Since the discover of hormone synthesis capacity, the adipose tissue has conquered a central role in the complex system of metabolism, hunger regulation, immunity responses and fertility [20]. In fact, the adypocite produces the adipose derived hormones: leptin [21,22] and adiponectin [22,23], playing a key role in regulating energy balance, while the adipocytokines (resistin, chemerin, visfatin, interleukin-6, plasminogen activator inhibitor-1, retinol binding protein 4 and tumor necrosis factor alpha, angiotensin) are also immunomodulating agents [24]; anyway, all these substances are implicated in developing metabolic syndrome [25]. Last, but not least, in the fat tissue estradiol derives from aromatase activity on testosterone [26]. Adipose cells bioactivity is under the control of both hormonal (insulin, cortisol) and nervous inputs (efferent vagus), and the tissue differentiation too has been influenced by genes and their transcriptional factors (peroxisome proliferator-activated receptor γ) and different signals, pro and anti-differentiation, which are provided by locally produced growth factors, cytokines, and circulating hormones (insulin, insuline-like growth factor 1 - IGF1, growth hormone - GH, thyroid hormones, glucocorticoids) [27,28].

Recently chemicals have been identified as one of the environmental factors that may affect obesity and associated diseases [29]. In particular, exposure to Bisphenol A (BPA), one of the chemicals that we incidentally intake, during the critical early life time period has been reported to determine the development of obesity and hyperlipidemia. More specifically, BPA has been shown to affect the glucose transport in adipocytes and at environmentally relevant doses to inhibit the release of a key adipokine that protects humans from metabolic syndrome [29-31].

In addition, several interesting associations between different phthalate metabolites and obesity outcomes have been reported [32]. These adverse human health effects seem to be related to an interaction of these chemicals with peroxisome proliferator-activated receptors (PPARs), members of the nuclear receptor superfamily [33]. Moreover, tributyltin (TBT) has been suggested to be one of the environmental chemicals that lead to excessive accumulation of adipose tissue [34].

In particular, exposure to EDC during critical periods of development may result in adverse health effects that may not be apparent until much later in life, including obesity and diabetes [12].

More specifically, prenatal exposure to the dichlorodiphenyl-dichloroethylene (DDE) seems to be able to contribute to the obesity epidemic in women [35] and reduced growth in early life has been related to insulin resistance [36]

More recently, alterations of adipogenesis, as well as disruption of the adiposity gene expression and leptin synthesis have been observed [37].

EDCs are also able to stimulate the glucocorticoid receptor (GR) and as glucocorticoid signaling is central to adipocyte differentiation, they are able to promote adipogenesis in the 3T3-L1 Cell Line through the activation of the GR, thus leading to obesity [38].

A key role seems to play adiponectin, an adipocyte-specific hormone that increases insulin sensitivity and reduces tissue inflammation. As a consequence, any factor that suppresses adiponectin release could lead to insulin resistance and increased susceptibility to obesity and its associated diseases [31].

Furthermore, it has been shown that 2,3,7,8-tetrachlorodibenzo-p-dioxin (TCDD) induces complex changes in enzymes of oxidative stress in adipocytes and may cause insulin resistance [39,40].

## Conclusions

Obesity has a clearly remarkable impact on health related quality of life. However, the determinants of obesity pandemic are many and yet poorly defined.

There is emerging evidence that exposure to environmental chemicals is associated with obesity and related diseases, even if a cause-effect relationship between the two events has still to be demonstrated. As a consequence, further studies need to be carried out (i) to perform updated exposure measures for hazard and risk estimation, (ii) to define the assessment of the potential hazards emanating from these chemicals, and (iii) as no risk may be justified when the health of humans is involved, to find alternative and better quality materials replacing those at this moment present on the market.

## Abbreviations

BPA: Bisphenol A; DDE: dichlorodiphenyl-dichloroethylene; EDC: endocrine disrupting chemicals; IGF - 1: insuline-like growth factor - 1; GH: growth hormone; GR: glucocorticoid receptor; PBDEs: polybrominated diphenyl ethers; PCBs: polychlorinated biphenyls; POPs: persistent organic pollutants; PPARs: peroxisome proliferator-activated receptors; TBT: tributyltin; TCDD: 2,3,7,8-tetrachlorodibenzo-p-dioxin
